# Groundnut Bud Necrosis Virus Modulates the Expression of Innate Immune, Endocytosis, and Cuticle Development-Associated Genes to Circulate and Propagate in Its Vector, *Thrips palmi*

**DOI:** 10.3389/fmicb.2022.773238

**Published:** 2022-03-17

**Authors:** Deepak Kumar Mahanta, Sumit Jangra, Priti, Amalendu Ghosh, Parva Kumar Sharma, Mir Asif Iquebal, Sarika Jaiswal, Virendra Kumar Baranwal, Vinay Kumari Kalia, Subhas Chander

**Affiliations:** ^1^Insect Vector Laboratory, Advanced Centre for Plant Virology, ICAR-Indian Agricultural Research Institute, New Delhi, India; ^2^Division of Entomology, ICAR-Indian Agricultural Research Institute, New Delhi, India; ^3^Center for Agricultural Bioinformatics, ICAR-Indian Agricultural Statistics Research Institute, New Delhi, India; ^4^ICAR- National Research Centre for Integrated Pest Management, New Delhi, India

**Keywords:** melon thrips, tospovirus, virus–vector relationship, RT-qPCR, transcriptome

## Abstract

*Thrips palmi* (Thysanoptera: Thripidae) is the predominant tospovirus vector in Asia-Pacific region. It transmits economically damaging groundnut bud necrosis virus (GBNV, family *Tospoviridae*) in a persistent propagative manner. Thrips serve as the alternate host, and virus reservoirs making tospovirus management very challenging. Insecticides and host plant resistance remain ineffective in managing thrips–tospoviruses. Recent genomic approaches have led to understanding the molecular interactions of thrips–tospoviruses and identifying novel genetic targets. However, most of the studies are limited to *Frankliniella* species and tomato spotted wilt virus (TSWV). Amidst the limited information available on *T. palmi*–tospovirus relationships, the present study is the first report of the transcriptome-wide response of *T. palmi* associated with GBNV infection. The differential expression analyses of the triplicate transcriptome of viruliferous vs. nonviruliferous adult *T. palmi* identified a total of 2,363 (1,383 upregulated and 980 downregulated) significant transcripts. The Gene Ontology (GO) and Kyoto Encyclopedia of Genes and Genomes (KEGG) pathway enrichment analyses showed the abundance of differentially expressed genes (DEGs) involved in innate immune response, endocytosis, cuticle development, and receptor binding and signaling that mediate the virus invasion and multiplication in the vector system. Also, the gene regulatory network (GRN) of most significant DEGs showed the genes like *ABC transporter*, *cytochrome P450*, *endocuticle structural glycoprotein*, *gamma-aminobutyric acid (GABA) receptor*, *heat shock protein 70*, *larval and pupal cuticle proteins*, *nephrin*, *proline-rich protein*, *sperm-associated antigen*, *UHRF1-binding protein*, *serpin*, *tyrosine–protein kinase receptor*, etc., were enriched with higher degrees of interactions. Further, the expression of the candidate genes in response to GBNV infection was validated in reverse transcriptase-quantitative real-time PCR (RT-qPCR). This study leads to an understanding of molecular interactions between *T. palmi* and GBNV and suggests potential genetic targets for generic pest control.

## Introduction

Thrips are minute, fringed winged insects that belong to the family Thripidae in the order Thysanoptera. Globally, thrips are considered as one of the most economically damaging pests of a wide range of food, feed, and fiber crops (Riley et al., 2011). Thrips rasp the soft plant tissues and suck plant sap from leaves, stems, flowers, and fruits. The infested plant parts develop distinctive silvery or bronze scarring. High incidence leads to curling, rolling, folding, distortion of leaves, and deformation of fruits. Besides, thrips are sole transmitters of tospoviruses (genus *Orthotospovirus*, family *Tospoviridae*, and order *Bunyavirales*) that cause significant economic losses to crop production across the globe (Ullman et al., 1992, 1997; Whitfield et al., 2005; Oliver and Whitfield, 2016). Annual losses due to tospovirus outbreaks are estimated to cost growers over US$ 1 billion worldwide (Pappu et al., 2009). To date, 16 thrips species are reported to vector 29 tospoviruses (Rotenberg et al., 2015; Ghosh et al., 2017). Tospoviruses propagate within thrips and thrips serve as effective alternate hosts and virus reservoirs which make tospovirus management very difficult (Ullman et al., 2002; Whitfield et al., 2005).

Melon thrips (*Thrips palmi* Karny) is a predominant thrips species in Asia and has spread to the Pacific, Florida, Hawaii, the Caribbean, South America, West Africa, and Australia (Bhatti, 1980; Johnson, 1986; Pantoja et al., 1988; Cooper, 1991; Palmer, 1992; Monteiro et al., 1995; Guyot, 1998; Kawai, 2001; Cannon et al., 2007; Ghosh et al., 2020). It infests over 200 plant species of family Asteraceae, Cucurbitaceae, Fabaceae, and Solanaceae (Walker, 1992; Cermeli and Montagne, 1993). *T. palmi* is known to transmit seven species of tospoviruses, such as calla lily chlorotic spot virus (CCSV), capsicum chlorosis virus (CaCV), groundnut bud necrosis virus (GBNV), melon yellow spot virus (MYSV), tomato necrotic ringspot virus (TNRV), watermelon bud necrosis virus (WBNV), and watermelon silver mottle virus (WSMoV; Ghosh et al., 2017, 2020). GBNV is the predominant tospovirus in Asia causing annual losses of approximately US$ 89 million. As much as 70%–90% of losses in groundnut were recorded due to infection of GBNV in India (Singh and Srivastava, 1995). Outbreaks of GBNV in tomatoes caused up to 100% disease incidence from 2003 to 2006 in India (Kunkalikar et al., 2011). Yield losses up to 29% have been reported in potatoes due to GBNV infection (Singh et al., 1997). Several management strategies have been adopted around the world, with insecticides and host plant resistance being the core components for thrips and tospovirus management, respectively. Farmers worldwide spend more than US$ 50 million annually to manage this vector. Insecticides are largely ineffective due to the emergence of resistant thrips population. Genetic host plant resistance is not available for all the crop species that are infected by thrips–tospoviruses. Understanding the interactions between thrips and tospovirus will be helpful to identify novel genetic targets to interrupt the interrelationship and restrict the epidemics of thrips–tospoviruses.

Various genomic approaches have been reported to understand the molecular interactions of thrips and tospoviruses. However, most of these studies are focused on tomato spotted wilt virus (TSWV) and *Frankliniella* species (Badillo-Vargas et al., 2012; Zhang et al., 2013; Stafford-Banks et al., 2014; Schneweis et al., 2017; Han and Rotenberg, 2021). *Frankliniella occidentalis* genes associated with host defense, insect cuticle structure and development, metabolism, and transport are regulated by TSWV infection (Schneweis et al., 2017). Genes associated with intracellular transport, development, and immune responses are regulated by TSWV infection in *Frankliniella fusca* (Shrestha et al., 2017). Transcriptome-wide responses of *T. palmi* associated with CaCV infection revealed upregulation of innate immune, digestion of proteins, and lipids-related genes, while the genes associated with the structural constituent of the cuticle were downregulated (Gamage et al., 2018). Tospovirus (WBNV) infects the epithelial cells of the anterior midgut of *T. palmi* and spreads to primary salivary glands *via* connecting ligaments and other parts of the alimentary canal (Ghosh et al., 2021). Tospovirus (WSMoV) propagates in the primary salivary glands and midgut of *T. palmi* (Mou et al., 2021). GBNV infection was found to negatively alter the survivability, adult longevity, and reproductive potential of *T. palmi* (Ghosh et al., 2019). Host plant also influences the biology and transmission of tospovirus (WBNV) by *T. palmi* (Dhall et al., 2021). Little is known about the differentially expressed genes (DEGs), pathways, and molecular interactions of *T. palmi* in response to GBNV infection. The present study reports the candidate genes of *T. palmi* responsive to GBNV infection through a comparative transcriptomic approach that would be novel targets for generic management of the thrips–tospovirus complex.

## Materials and Methods

### Establishing an Isofemale Population of *Thrips palmi*

An isofemale population of *T. palmi* maintained at Advanced Centre for Plant Virology, Indian Agricultural Research Institute (IARI), New Delhi, was used in the study. The identification of *T. palmi* was based on the morphological keys and mitochondrial cytochrome oxidase subunit I (mtCOI) sequences amplified in PCR using primer pairs, LCO 1490 and HCO 2198 (Folmer et al., 1994; Jangra et al., 2020). The population was established from a single adult female of *T. palmi* on eggplant (var. Navkiran, Mahyco). The population has been maintained at the thrips rearing facility under controlled environmental conditions at 28 ± 1°C temperature, 60 ± 10% relative humidity, and 16 h of light–8 h of the dark since 2018. Fresh virus-free healthy eggplants were supplied as and when required. The isofemale population has been used in replicate throughout the experiment.

### Establishment of Pure Culture of GBNV

The initial inoculum of GBNV was collected from the pure culture maintained at Advanced Centre for Plant Virology, IARI. Healthy cowpea plants (var. Pusa Komal) were raised from seeds in a plant growth chamber (A2000, Conviron, Canada). The healthy cowpea plants at a two-leaf stage were sap-inoculated with GBNV as described by Ghosh et al. (2021). Briefly, the sap was extracted by grinding the inoculum in a pre-sterilized mortar on ice. Ice-cold sodium phosphate buffer (0.01 M, pH 7.0) was added at tissue to buffer ratio of 1:6 wt/vol. β-mercaptoethanol (0.2%) was added while grinding the tissue. A pinch of Celite was dusted on the cowpea plants. The sap was applied to the leaves by gently rubbing with gloved hands. The inoculated plants were washed with sterile distilled water after a while. The inoculated plants were maintained at 25 ± 1°C, 60 ± 10% relative humidity, and 16 h of light–8 h of dark under insect-proof conditions. Nutrient solution containing macro, meso, and trace elements at appropriate concentrations was provided to plants and regularly monitored for symptom appearance. All the inoculated plants were tested in reverse-transcriptase PCR (RT-PCR) using GBNV-specific primers, AG109F and AG110R (Supplementary Table 1) as described below.

### Diagnosis of GBNV Infection by RT-PCR

The total RNA was isolated from cowpea plants using RNeasy Plant Mini Kit (Qiagen, Germantown, MD, United States). Complementary DNA (cDNA) was synthesized with random primers using FIREScript RT cDNA synthesis kit (Solis Biodyne, Estonia). The reaction mixture contained 1X RT reaction buffer, 1.0 μg template RNA, 5 μM random primers, 2 mM dNTP mix, 10-unit FIREScript RT, and 20-unit RiboGrip RNase inhibitor. The reverse transcription was carried out in a T100 thermocycler (Bio-Rad, United States) by primer annealing at 25°C for 10 min, reverse transcription at 50°C for 30 min and followed by enzyme inactivation at 85°C for 5 min. PCR was carried out in 25 μl reactions containing 2 μl cDNA, 2.5 μl 10X PCR buffer (Thermo Fischer Scientific), 0.4 μM each of forward and reverse primers, 260 μM dNTP mix (Thermo Fischer Scientific), and 2 U Dream*Taq* polymerase (Thermo Fisher Scientific). PCR was performed in a T100 Thermal Cycler with 95°C for 5 min followed by 30 cycles of 94°C for 30 s, 59°C for 45 s, and 72°C for 1 min followed by a final extension at 72°C for 7 min. RT-PCR products were resolved on 1% agarose gel stained with GoodView (BR Biochem, India) and observed in a gel documentation system (MasteroGen Inc., Taiwan). The purified product was sequenced bidirectional. The sequences were processed by BioEdit, and BLASTn was performed to check the species homology. The consensus sequence was submitted to GenBank.

### Generation of Viruliferous and Nonviruliferous *Thrips palmi* Population

The freshly emerged first instar larvae (L1, less than 24 h old) of *T. palmi* were collected using a Camel hairbrush from the isofemale population on eggplants and used for GBNV acquisition in three replicates to generate the viruliferous population. The virus acquisition setup was established as described by Ghosh et al. (2021). About 50 *T. palmi* L1s in each replicate were placed on a GBNV-infected leaf and allowed to feed for 24 h at 28 ± 1°C and 60 ± 10% relative humidity. The setup was continuously monitored to confirm the feeding of larvae on the leaf. After the acquisition access period, the larvae were transferred on a detached healthy cowpea leaf. The petiole of the detached leaf was inserted in a slant of 0.8% dextrose agar within an insect breeding dish (10 cm diameter, 4 cm height, SPL Life Sciences, Korea). The GBNV-exposed *T. palmi* were reared up to the adult stage on cowpea leaves at 28 ± 1°C, 60 ± 10% relative humidity, and 16 h of light–8 h of dark. The breeding dishes were regularly monitored and supplied with fresh leaves as and when required. To generate nonviruliferous *T. palmi* adults, the L1s were released on the healthy cowpea leaves placed on the agar slant within the insect breeding dishes in three replicates as described above. Adults were collected immediately after their emergence and used in the study. Three sets of viruliferous (designated as TpTrI1, TpTrI2, and TpTrI3) and nonviruliferous (TpTrH1, TpTrH2, and TpTrH3) *T. palmi* adult populations were developed. Each set was divided into two parts. One part was used for transcriptome sequencing and another part was preserved at −80°C for gene expression analysis in reverse transcriptase-quantitative real-time PCR (RT-qPCR).

Infection of GBNV in *T. palmi* was confirmed by randomly collecting five individual adults from each of the viruliferous and nonviruliferous insect populations and testing in RT-PCR. Total RNA from adults of *T. palmi* was isolated using NucleoSpin RNA XS (Macherey-Nagel, Germany), and RT-PCR was performed as described above.

### RNA Extraction, Library Preparation, and Sequencing

Three sets of viruliferous (TpTrI1, TpTrI2, and TpTrI3) and nonviruliferous (TpTrH1, TpTrH2, and TpTrH3) adults of *T. palmi* were subjected to total RNA isolation using TRIzol (Invitrogen, United States). Each set comprised 10 adult individuals that included both females and males (1:1).

RNA quality was checked using RNA 6000 Nano Kit (Agilent Technologies, United States) on 2100 Bioanalyzer (Agilent Technologies) with a minimum RNA Integrity Number (RIN) value of 7. RNA concentrations were determined with a NanoDrop ND-8000 spectrophotometer (Thermo Fischer Scientific). RNA-Seq libraries for all samples were prepared using NEBNext UltraII RNA library preparation kit for Illumina (New England Biolabs, United States), and sequencing was done in a single HiSEQ 4,000 (Illumina, Inc. United States) lane using 150 bp paired-end chemistry. The library preparation and sequencing were done by commercial service providers (NxGen Bio Life Sciences, India). Briefly, total RNA was used to purify poly(A) messenger RNA (mRNA) using oligo-dT beads. Magnetic beads were used for two rounds of purification. During the second elution of the poly-A RNA, the RNA was also fragmented into 200–500 bp pieces in the presence of divalent cations at 94°C for 5 min using an ultrasonicator. The cleaved RNA fragments were copied into first-strand cDNA using SuperScript-II Reverse Transcriptase (Thermo Fischer Scientific) and random primers. After second-strand cDNA synthesis, fragments were end-repaired and A-tailed, and indexed adapters were ligated. The products were purified and enriched with PCR to create the final cDNA library. The tagged cDNA libraries were pooled in equal ratios and used for 2 × 150 bp paired-end sequencing on a single lane of the Illumina HiSeq4000. Illumina clusters were generated and were loaded onto Illumina Flow Cell on Illumina HiSeq 4000 instrument and sequencing was carried out. After sequencing, the samples were demultiplexed and the indexed adapter sequences were trimmed using the CASAVA v1.8.2 software (Illumina, Inc.).

### Data Preprocessing, Assembly, and Differential Gene Expression

The obtained low-quality raw reads were preprocessed to remove the adaptor contamination using Trim Galore v0.4.1^1^ with the quality score <20%, and trimming ambiguous “*N*” nucleotides with a ratio of “*N*” > 5%. A reference genome index was established using BWAv0.7.5 (Li and Durbin, 2009). The processed clean paired-end reads were used to map the reference genome of *T. palmi* (Guo et al., 2020) by BWA-MEM (Li, 2013). Read numbers mapped to every gene were counted using Samtools v0.1.19 software. Differential expression between the viruliferous and nonviruliferous *T. palmi* populations was analyzed using the DESeq R package.^2^ An absolute value of log_2_ fold change >2 and value of *p* <  0.05 were used as the thresholds to judge the significance of gene expression differences.

### GO and KEGG Pathway Enrichment of Differentially Expressed Genes

Gene annotations and functional enrichment analysis including Gene Ontology (GO) and Kyoto Encyclopedia of Genes and Genomes (KEGG) biological pathways were performed to identify the DEGs that were significantly enriched in GO terms or biological pathways post-GBNV infection. GO enrichment analysis for the identified GO terms was performed using the Fisher’s exact test available in the Blast2Go program (Conesa and Götz, 2008). Gene annotations against the Uniprot GO database^3^ were performed by aligning DEGs to the NR database using Blast2Go v5.2.5 software. GO terms were also assigned using the QuickGO online search tool.^4^ KEGG pathway enrichment analysis of DEGs was performed using the KEGG database resource^5^ to identify the pathways that were differentially regulated between viruliferous and nonviruliferous *T. palmi* with corrected value of *p* < 0.05. KEGG pathway enrichment was performed by calculating the enrichment factor for all the identified KEGG pathways. The top 20 highly enriched pathways were plotted based on the rich factor.

### Gene Regulatory Network Analysis

The top 20 genes each from up- and downregulated DEGs were selected for the construction of gene regulatory networks (GRNs). These DEGs were filtered based on the log fold change values. The network was analyzed and visualized using Cytoscape v3.7.2, where gene correlation was computed using the Pearson’s correlation coefficient of the normalized expression values (Shannon et al., 2003). Network Analyzer plug-in was used to estimate the network centrality and topology.

### Validation of RNA-Seq Expression With Real-Time RT-qPCR

To validate the differential gene expression data by RNA-Seq analysis, a few highly up- and downregulated DEGs of *T. palmi* were selected to assess the gene expression in RT-qPCR. A part of the preserved viruliferous and nonviruliferous *T. palmi* populations was used in RT-qPCR analysis. Initially, three sets of primers were designed for each of the target genes, and finally, one pair of primers for each gene was selected after validation. *β-tubulin* was considered as an endogenous control. All the primer sets were validated and optimized in a gradient PCR. Following thermal cycling conditions were followed for validation and optimization of target genes and endogenous control primers: initial denaturation at 95°C for 5 min, then 35 cycles of 95°C for 40 s, 50°C–60°C (depending upon the Tm of primer pairs) for 40 s, and 72°C for 40 s followed by a final extension at 72° C for 10 min. The primer pairs that produced a single specific sharp band at the same PCR conditions for *β-tubulin* primers were further selected for RT-qPCR experiment. The list of primers that were validated and used in RT-qPCR assay is included in Supplementary Table 1.

The relative expressions of target genes were estimated by RT-qPCR following 2^−ΔΔCT^ method (Livak and Schmittgen, 2001). Firstly, total RNA was isolated from viruliferous (TpTrI1, TpTrI2, and TpTrI3) and nonviruliferous (TpTrH1, TpTrH2, and TpTrH3) thrips samples stored at −80°C using NucleoSpin RNA XS. cDNA was synthesized with oligo (dT) primer using FIREScript RT cDNA synthesis kit. The reaction mixture contained 1X RT reaction buffer, 1.0 μg template RNA, 5 μM oligo dT primer, 2 mM dNTP mix, 10-unit FIREScript RT, and 20-unit RiboGrip RNase inhibitor. The reverse transcription was carried out in a T100 thermocycler at 50°C for 30 min followed by enzyme inactivation at 85°C for 5 min. RT-qPCR was performed in an Insta Q 48M (Himedia, India) using the DyNAmo ColorFlash SYBR Green qPCR Kit (Thermo Fisher Scientific). A 20 μl of RT-qPCR reaction mixture consisted of 10 μl of 1X DyNAmo ColorFlash SYBR Green Master Mix, 0.25 μM ROX passive reference dye, 0.2 μM each forward and reverse primer, and 2 μl template cDNA. Thermal cycling was performed as initial denaturation at 95°C for 5 min, 35 cycles of 95°C for 40 s, annealing at 53–59°C (depending upon the Tm of primer pairs) for 40 s, and 72°C for 40 s. Since SYBR Green I dye binds nonspecifically to any double-stranded DNA, a dissociation or melting stage was added after every reaction to determine the specificity of the amplicons based on the melting curves. The RT-qPCR was performed with three biological and two technical replicates. Log_2_ fold change value was calculated and relative expression of mRNA was determined by normalized log_2_ 2^−ΔΔCT^ (Livak and Schmittgen, 2001) values of viruliferous (TpTrI) in comparison to nonviruliferous (TpTrH) *T. palmi*. The ΔC_T_ was calculated as C_T_ of targeted gene—C_T_ of endogenous control. ΔΔCT value was calculated as average ΔC_T_ of nonviruliferous replicates—ΔCT of each replicate. The average 2^-(ΔΔCT)^ was calculated for both the viruliferous and nonviruliferous populations and transformed to log_2_ 2^-(ΔΔCT)^. The log_2_-fold change of the target gene expression in viruliferous *T. palmi* population was estimated by normalizing the fold change values of the nonviruliferous population. The relative expressions of target genes post-GBNV exposure in RT-qPCR assay were compared with log_2_-fold changes obtained through RNA-Seq analysis, and Pearson’s correlation coefficient was calculated using CORREL function in MS Excel.

## Results

### *Thrips palmi* Population

The isofemale *T. palmi* population generated from a single adult female was confirmed on the basis of morphological characters. The adults of *T. palmi* were yellow in color measuring about 0.8 mm–1 mm in length. Three brick red ocelli were visible in a triangular fashion between the compound eyes. A pair of interocellar setae originated outside the ocellar triangle. The head was quadrangular in shape with seven segmented antennae. Female adults were comparatively larger than males. Female adults had sharp ovipositor at the apex of the abdomen while males were with rounded apex. Further, the nucleotide sequence of mtCOI confirmed the identity as *T. palmi*. A 657 bp fragment of mtCOI was amplified using primer pairs LCO 1490 and HCO 2198 (Folmer et al., 1994). The nucleotide sequence of the amplified product showed 100% homology to other *T. palmi* isolates (MN594549, NC_039437, and KX622379) available in GenBank. The sequence can be retrieved from the GenBank with the Accession number MW020349.

### GBNV Infection of *T. palmi* and Cowpea Plant

The initial inoculum of GBNV was collected from the pure culture (tomato isolate) maintained at Advanced Centre for Plant Virology, IARI. Healthy cowpea plants (*Vigna unguiculata* var. Pusa Komal) were sap-inoculated with GBNV. Characteristic symptoms of GBNV were recorded on sap-inoculated cowpea plants 10–14 days post-inoculation (dpi). Local lesions appeared on inoculated leaves and yellow necrotic spots with multiple rings-like structures were visible on systemic leaves. RT-PCR with GBNV-specific primers produced an amplicon of 1.8 kb for all symptomatic samples that confirmed the GBNV infection in inoculated plants. No amplification was observed in RT-PCR for healthy plant samples. Further, the nucleotide sequence of the amplified product showed more than 97% identity to GBNV. The sequence can be retrieved from the GenBank with Accession No. MN566913.

The isofemale *T. palmi* adults in three replicates (TpTrI1, TpTrI2, and TpTrI3) exposed to GBNV-infected cowpea plants during the L1 stage were tested in RT-PCR. A 1.8 kb product was visualized on agarose gel that confirmed the infection of GBNV in *T. palmi* adults. *T. palmi* populations (TpTrH1, TpTrH2, and TpTrH3) that were exposed to healthy cowpea plants did not produce any GBNV-specific amplification in RT-PCR.

### Data Preprocessing, Assembly, and Differential Expression Analysis

*T. palmi* transcriptome data generated a total of 156,127,441 raw reads of 150 bp from three replicated cDNA libraries of each viruliferous and nonviruliferous *T. palmi* population. Around 46.84 Gb of the sequence was generated. The average reads per library were about 26,021,240 (Supplementary Table 2). After preprocessing, a total of 156,074,069 clean reads of 20–150 bp were filtered from all six libraries. The preprocessed reads ranged between 99.95 and 99.98%. After removing the low-quality reads from all the sets, the high-quality reads were used for further analyses. These were mapped with the *T. palmi* genome (Guo et al., 2020) yielding an average mapping percent of 97.31%.

**Figure 1:**
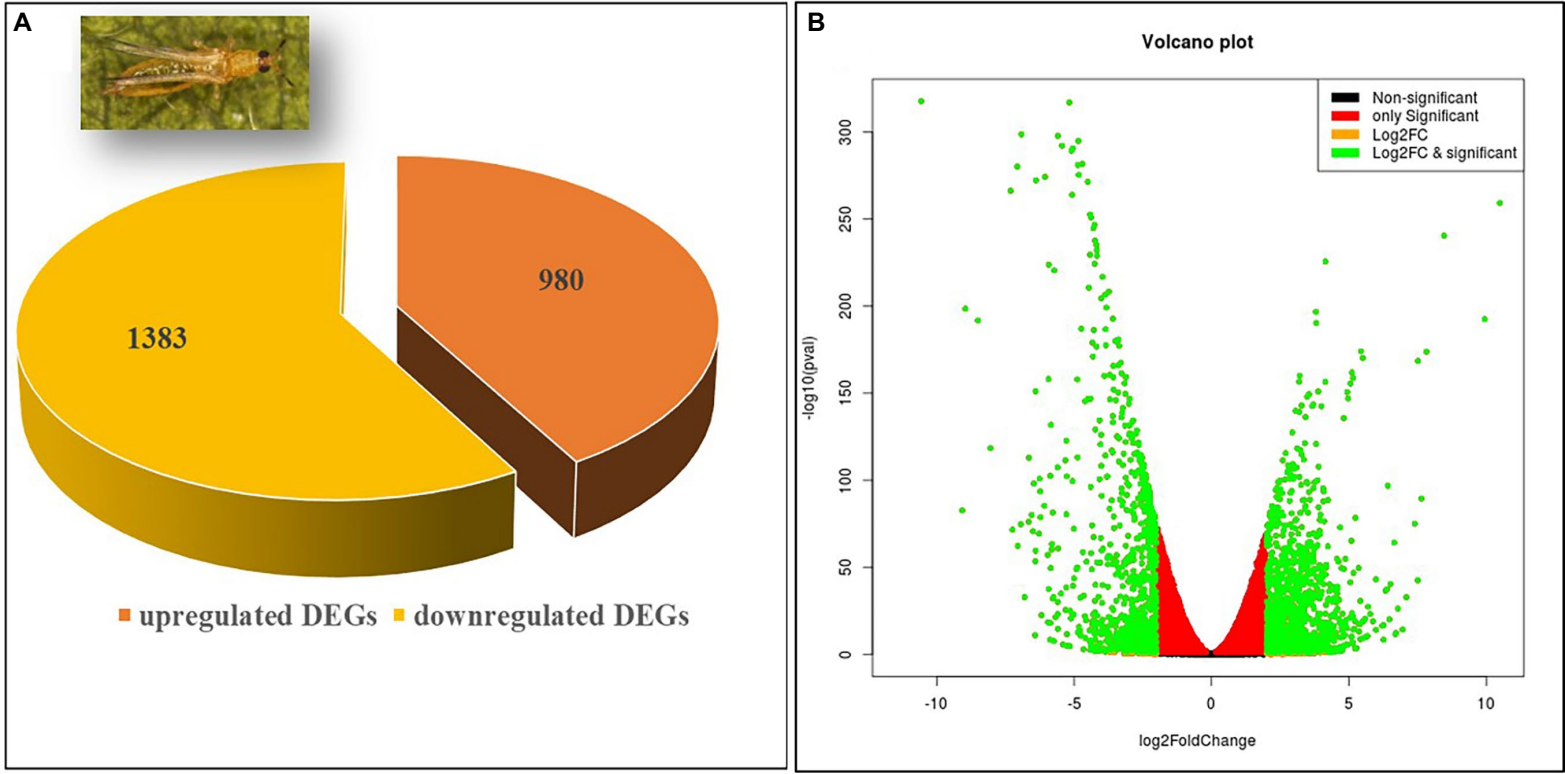
The proportion of differentially expressed genes (DEGs) in RNA-Seq analysis. **(A)** A total of 1,383 DEGs were found upregulated while 980 were downregulated. **(B)** Volcano plot of DEGs. The *x* axis shows the fold change in gene expression between different samples, and the *y* axis shows the statistical significance of the differences. Significantly up- and downregulated genes with log_2_ FC ≥ 2 are highlighted in green. The black dots represent insignificant differentially expressed genes.

The differential expression analysis identified a total of 28,165 transcripts in the viruliferous vs. nonviruliferous *T. palmi* populations. Out of these, a total of 2,363 transcripts were significantly expressed in viruliferous *T. palmi* at stringent filtering parameters (log_2_ fold change > ± 2 and adjusted value of *p* < 0.05). A total of 1,383 differentially expressed transcripts were upregulated, while 980 transcripts were downregulated in viruliferous (pooled TpTrI) as compared to nonviruliferous (pooled TpTrH) populations (Figure 1). Table 1 represents the top 20 up- and downregulated genes of *T. palmi* under the stringent filtering criteria in response to GBNV-infection. The putative genes of *T. palmi* such as *cytochrome P450* (*CYP*), *gamma-aminobutyric acid* (*GABA*) *receptor*, *heat shock protein* (*hsp70*), *nephrin*, *sperm-associated antigen 6* (*spag6*)*-like*, and *ubiquitin-like containing PHD* and *RING finger domains 1* (*UHRF*-*1*)*-binding protein 1*, etc. were found highly upregulated after exposure to GBNV. Significant downregulations of *ABC transporter*, *arrestin domain-containing 3* (*ARRDC3*), *elongation of very-long-chain fatty acid* (*ELOVL*), *dynamin-1-like*, *serpin H1-like*, *tyrosine–protein kinase receptor Tie-1* (*TIE1*), *larval cuticle protein A2B-like*, *pupal cuticle protein C1B-like*, and *endocuticle structural glycoprotein SgAbd-2-like*, etc., were also observed.

**Table 1:** Top upregulated and downregulated genes of *Thrips palmi* in response to GBNV infection.

S. No	Name of the gene	Accession	GO term
**Upregulated DEGs**			
1	*Calphotin-like isoform X2*	XM_034379643	Epithelium-like organization (GO:0140509), identical protein binding (GO:0042802), and skein-like inclusion (GO:0097420). Regulate the amount of free cytoplasmic calcium. Rhabdomere development and photoreceptor cell survival (Martin et al., 1993)
2	*Proline-rich protein 36-like isoform X1*	XM_034379634	Proline-rich region binding (GO:0070064), protein peptidyl-prolyl isomerization (GO:0000413), and ubiquitin-like protein binding (GO:0032182)
3	*LOW-QUALITY PROTEIN: probable dipeptidyl-aminopeptidase B*	XM_034387198	Cytoplasm protein quality control (GO:0140455), aminopeptidase activity (GO:0004177), cysteine-type aminopeptidase activity (GO:0070005), and serine-type aminopeptidase activity (GO:0070009)
4	*Sodium channel protein para isoform X8*	XM_034385472	Sodium channel activity (GO:0005272), sodium channel complex (GO:0034706), and protein transmembrane transporter activity (GO:0008320)
5	*Nephrin-like*	XM_034394430	Epithelium-like (GO:0140509), skein-like inclusion (GO:0097420), X11-like protein binding (GO:0042988), and Toll-like receptor binding (GO:0035325)
6	*cAMP-specific 3',5'-cyclic phosphodiesterase isoform X1*	XM_034385045	3′,5′-cyclic-AMP phosphodiesterase activity (GO:0004115), phosphoric diester hydrolase activity (GO:0008081), and 3′,5′-cyclic diguanylic acid metabolic process (GO:0052653)
7	*UHRF1-binding protein 1-like isoform X3*	XM_034383841	X11-like protein binding (GO:0042988), ubiquitin-like protein binding (GO:0032182), and identical protein binding (GO:0042802)
8	*Protein furry-like isoform X1*	XM_034399231	Identical protein binding (GO:0042802), X11-like protein binding (GO:0042988), ubiquitin-like protein binding (GO:0032182), Sm-like protein family complex (GO:0120114), and fasciclin-like arabinogalactan protein metabolic process (GO:0010408)
9	*SCY1-like protein 2 isoform X3*	XR_004587416	Identical protein binding (GO:0042802), X11-like protein binding (GO:0042988), ubiquitin-like protein binding (GO:0032182), Toll-like receptor 2 binding (GO:0035663), Sm-like protein family complex (GO:0120114), and toll-like receptor 2 signaling pathway (GO:0034134)
10	*Gamma-aminobutyric acid receptor subunit beta isoform X8*	XR_004588251	Gamma-aminobutyric acid receptor clustering (GO:0097112), gamma-aminobutyric acid secretion (GO:0014051), gamma-aminobutyric acid transport (GO:0015812), GABA receptor complex (GO:1902710), and gamma-aminobutyric acid transmembrane transporter activity (GO:0015185)
11	*Sperm-associated antigen 6-like*	XM_034382859	TAP complex (GO:0042825), sperm motility (GO:0097722), sperm capacitation (GO:0048240), and sperm flagellum (GO:0036126)
12	*Proteoglycan 4-like isoform X4*	XM_034375036	Proteoglycan binding (GO:0043394), Toll-like receptor 4 binding (GO:0035662), and toll-like receptor 4 signaling pathway (GO:0034142)
13	*Sodium channel protein para isoform X5*	XM_034385468	Sodium channel activity (GO:0005272), sodium channel complex (GO:0034706), sodium channel regulator activity (GO:0017080), and regulation of voltage-gated sodium channel activity (GO:1905150)
14	*Laccase-5*	XM_034375390	Oxidoreductase activity, acting on diphenols and related substances as donors, oxygen as acceptor (GO:0016682), hydroquinone: oxygen oxidoreductase activity (GO:0052716), MDA-5 binding (GO:0039556), laminin-5 complex (GO:0005610), and rhombomere 5 development (GO:0021571)
15	*Cytochrome P450 4C1-like*	XM_034387022	Retinoic acid 4-hydroxylase activity (GO:0008401), arachidonic acid 11,12-epoxygenase activity (GO:0008405), and coumarin 7-hydroxylase activity (GO:0008389)
16	*Serine protease snake-like*	XM_034378873	Serine-type peptidase activity (GO:0008236), serine protease inhibitor complex (GO:0097180), ubiquitin-like protein-specific protease activity (GO:0019783), regulation of serine-type peptidase activity (GO:1902571), and serine transport (GO:0032329)
17	*Phosphoribosylformylglycinamidine synthase*	XM_034387894	Phosphoribosylformylglycinamidine synthase activity (GO:0004642), phosphoribosylformylglycinamidine cyclo-ligase activity (GO:0004641), and hercynylselenocysteine synthase (GO:0044876)
18	*Lipase 3-like isoform X2*	XM_034393741	Lipase activity (GO:0016298), lipase binding (GO:0035473), toll-like receptor 3 signaling pathway (GO:0034138), and regulation of lipase activity (GO:0060191)
19	*Heat shock 70 kDa protein II-like*	XM_034395223	Heat shock protein binding (GO:0031072), protein refolding (GO:0042026), X11-like protein binding (GO:0042988), ubiquitin-like protein binding (GO:0032182), response to unfolded protein (GO:0006986), and heat dissipation (GO:0031653)
20	*Fatty acid synthase-like*	XM_034388838	Fatty acid synthase complex (GO:0005835), fatty acid synthase activity (GO:0004312), fatty acid binding (GO:0005504), fatty acid elongation (GO:0030497), and fatty acid homeostasis (GO:0055089)
**Downregulated DEGs**			
1	*Proline-rich protein HaeIII subfamily 1-like*	XM_034391864	Proline-rich region binding (GO:0070064), intramolecular proline-rich ligand binding (GO:0032840), ficolin-1-rich granule (GO:0101002), and protein peptidyl-prolyl isomerization (GO:0000413)
2	*Arrestin domain-containing protein 3-like*	XM_034389930	Arrestin family protein binding (GO:1990763), protein-containing complex (GO:0032991), RING-like zinc finger domain binding (GO:0071535), and RHG protein domain binding (GO:0089719)
3	*Mucin-2-like isoform X2*	XM_034396273	Mucin granule (GO:0098594), Toll-like receptor 2 binding (GO:0035663), and toll-like receptor 2 signaling pathway (GO:0034134)
4	*Basic salivary proline-rich protein 4-like*	XM_034378770	Proline-rich region binding (GO:0070064), intramolecular proline-rich ligand binding (GO:0032840), protein peptidyl-prolyl isomerization (GO:0000413), and procollagen-proline 4-dioxygenase complex GO:0016222
5	*Elongation of very-long-chain fatty acids protein AAEL008004-like*	XM_034386603	Very long-chain fatty acid catabolic process (GO:0042760), very long-chain fatty acid biosynthetic process (GO:0042761), and very long-chain fatty acid omega-hydroxylase activity (GO:0140692)
6	*Ubiquitin carboxyl-terminal hydrolase 36-like isoform X1*	XR_004588219	Ubiquitin-like hydrolase activity (GO:0140491), ubiquitin-like protein binding (GO:0032182), and ubiquitin-like protein transferase activity (GO:0019787)
7	*Serine/arginine repetitive matrix protein 1-like*	XM_034375486	Structural constituent of virion (GO:0039660), protein-arginine deiminase activity (GO:0004668), protein arginine phosphatase activity (GO:0098627), and protein arginine kinase activity (GO:1990424)
8	*Serpin H1-like*	XM_034391757	H1 histamine receptor binding (GO:0031807), serpin family protein binding (GO:0097655), epithelium-like organization (GO:0140509), and skein-like inclusion (GO:0097420)
9	*Myrosinase 1-like*	XM_034376507	Thioglucosidase binding (GO:0010180), thioglucosidase complex 1 (GO:0010169), toll-like receptor 1 signaling pathway (GO:0034130), and glucagon-like peptide 1 receptor activity (GO:0044508)
10	*Fidgetin-like protein 1 isoform X1*	XM_034396404	Toll-like receptor 1-Toll-like receptor 2 protein complex (GO:0035354), toll-like receptor 1 signaling pathway (GO:0034130), and glucagon-like peptide 1 receptor activity (GO:0044508)
11	*ABC transporter F family member 4-like*	XM_034380645	Sm-like protein family complex (GO:0120114), ABC-type transporter activity (GO:0140359)
12	*Peroxiredoxin-6-like*	XM_034396984	Peroxiredoxin activity (GO:0051920), toll-like receptor 6 signaling pathway (GO:0034150), and laminin-6 complex (GO:0005611)
13	*Alpha-tocopherol transfer protein-like*	XM_034375040	Alpha-tocopherol omega-hydroxylase activity (GO:0052871), vitamin E binding (GO:0008431), tocopherol cyclase activity (GO:0009976), and genetic transfer (GO:0009292)
14	*Tyrosine–protein kinase receptor Tie-1-like*	XM_034385734	Transmembrane receptor protein tyrosine kinase activity (GO:0004714), protein tyrosine kinase collagen receptor activity (GO:0038062), and transmembrane receptor protein tyrosine kinase signaling pathway (GO:0007169)
15	*Dynamin-1-like protein*	XM_034384104	DNM1L-mediated stimulation of mitophagy in response to mitochondrial depolarization (GO:0061735), Toll-like receptor 1-Toll-like receptor 2 protein complex (GO:0035354), and dynamin family protein polymerization involved in membrane fission (GO:0003373)
16	*Larval cuticle protein A2B-like*	XM_034397437	Structural constituent of chitin-based larval cuticle (GO:0008010), larval chitin-based cuticle development (GO:0008363), larval serum protein complex (GO:0005616), larval behavior (GO:0030537), and A2B adenosine receptor binding (GO:0031688)
17	*LOW-QUALITY PROTEIN: pupal cuticle protein C1B-like*	XM_034378539	Cytoplasm protein quality control (GO:0140455), pupal chitin-based cuticle development (GO:0008364), structural constituent of pupal chitin-based cuticle (GO:0008011), and pupal development (GO:0035209)
18	*Serine proteinase stubble-like*	XM_034381350	Proteinase activated receptor binding (GO:0031871), serine binding (GO:0070905), and serine transport (GO:0032329)
19	*Endocuticle structural glycoprotein SgAbd-2-like*	XM_034395161	Glycoprotein complex (GO:0090665), glycoprotein transport (GO:0034436), Toll-like receptor 2 binding (GO:0035663), N-glycan fucosylation (GO:0036071), protein deglycosylation (GO:0006517), and response to glycoprotein (GO:1904587)
20	*Phenoloxidase 2-like*	XM_034391057	Toll-like receptor 2 binding (GO:0035663), toll-like receptor 2 signaling pathway (GO:0034134)

### GO and KEGG Pathway Enrichment Analyses of Differentially Expressed Genes

**Figure 2:**
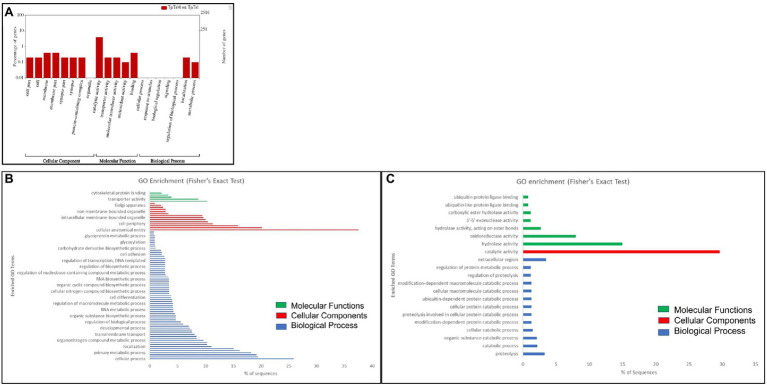
Gene ontology (GO) enrichment analysis of differentially expressed genes (DEGs). **(A)** DEGs were characterized under cellular components, molecular functions, and biological processes on the basis of GO analysis. GO of differential expressed **(B)** up- and **(C)** downregulated genes of viruliferous vs. nonviruliferous thrips populations. Green, red, and blue bars represent molecular functions, cellular components, and biological processes, respectively.

The 1,383 (58.52%) up- and 980 (41.48%) downregulated DEGs were categorized under the three major classes, namely, cellular components, biological processes, and molecular functions. The highest number of GO terms were categorized into molecular functions (70%) followed by cellular components (24%), and biological processes (6%; Figure 2A). In the molecular functions category, there was an abundance of genes associated with catalytic activity followed by binding, molecular transduction activity, antioxidant activity, and transporter activity. Similarly, genes associated with the membrane, membrane part, cell, cell part, protein-containing complex, organelle, synapse, synapse part were differentially enriched under the cellular components category. In the biological processes, genes involved in the cellular process, response to stimuli, signaling, regulation of the biological process, localization, metabolic process, biological regulation, and signaling were enriched. Within each of the functional groups, the top enrichment terms for both up- and downregulated DEGs identified as per Fisher’s exact test value of *p* are listed in Figures 2B,C, respectively.

**Figure 3:**
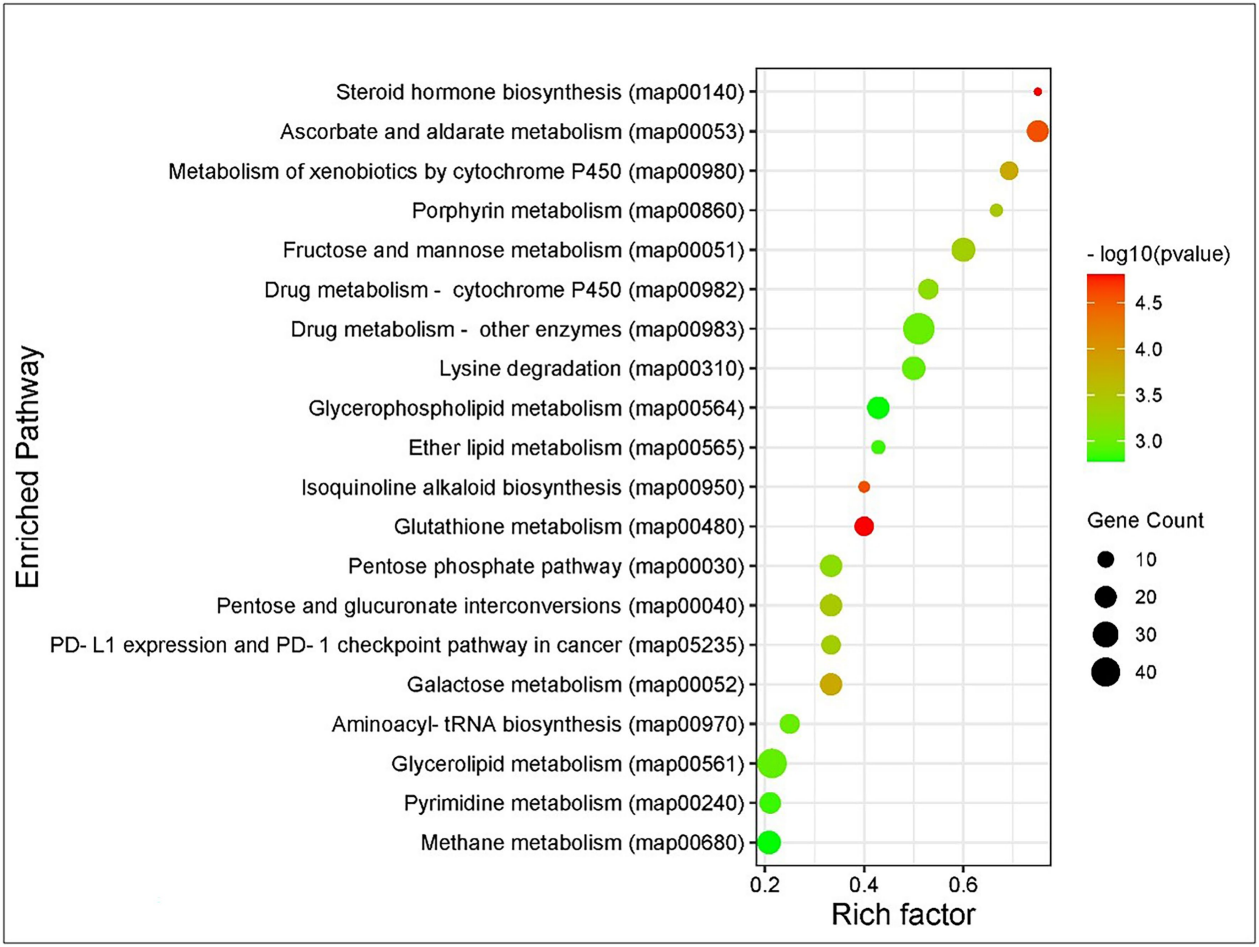
Kyoto Encyclopedia of Genes and Genomes (KEGG) pathway enrichment analysis of DEGs of *Thrips palmi* in response to groundnut bud necrosis virus (GBNV) infection. The rich factor is defined as the ratio of the number of DEGs annotated in a pathway to the number of all genes annotated in this pathway. *Y* axis indicates the pathway name; *x* axis indicates the enriched factor in each of the pathways. The bubble size indicates the number of DEGs. The color bar indicates the corrected value of *p*, the red represents a higher value, and the green represents a lower value.

Kyoto Encyclopedia of Genes and Genomes pathway analysis of DEGs showed that the genes were involved in the functions like metabolic pathways, biosynthesis of secondary metabolites, cellular processes, endocytosis, organismal systems, and signaling pathways. Majorly affected metabolic pathways were glycerolipid, glycerophospholipid, purine, fructose and mannose, galactose, glutathione, and carbon metabolism. The differentially expressed signaling pathways were calcium, cyclic adenosine monophosphate (cAMP), glucagon, hypoxia-inducible factor 1 (HIF-1), mitogen-activated protein kinase (MAPK), neurotrophin signaling, phospholipase D, Ras-proximate-1 (Rap1) signaling, and Ras signaling pathways. The most affected cellular and organismal systems were dopaminergic synapses, endocytosis, glutamatergic synapse, lysine degradation, neuroactive ligand-receptor interaction, oocyte meiosis, and synaptic vesicle cycle. In KEGG pathway enrichment analysis, the highest number of DEGs was annotated in drug metabolism (map00982, 00983) under the class xenobiotics biodegradation and metabolism followed by glycerolipid metabolism (map00561), methane metabolism (map00680), glycerophospholipid metabolism (map00564), and fructose and mannose metabolism (map00051; Figure 3). The pathway enrichment of DEGs showed the DEGs were significantly enriched in ascorbate and aldarate metabolism (map00053), steroid hormone biosynthesis (map00140), and metabolism of xenobiotics by cytochrome P450 (map00980) pathways. The pathways identified in GO enrichment analysis were consistent with the findings of the KEGG pathway study.

### Gene Regulatory Network Analysis

**Figure 4:**
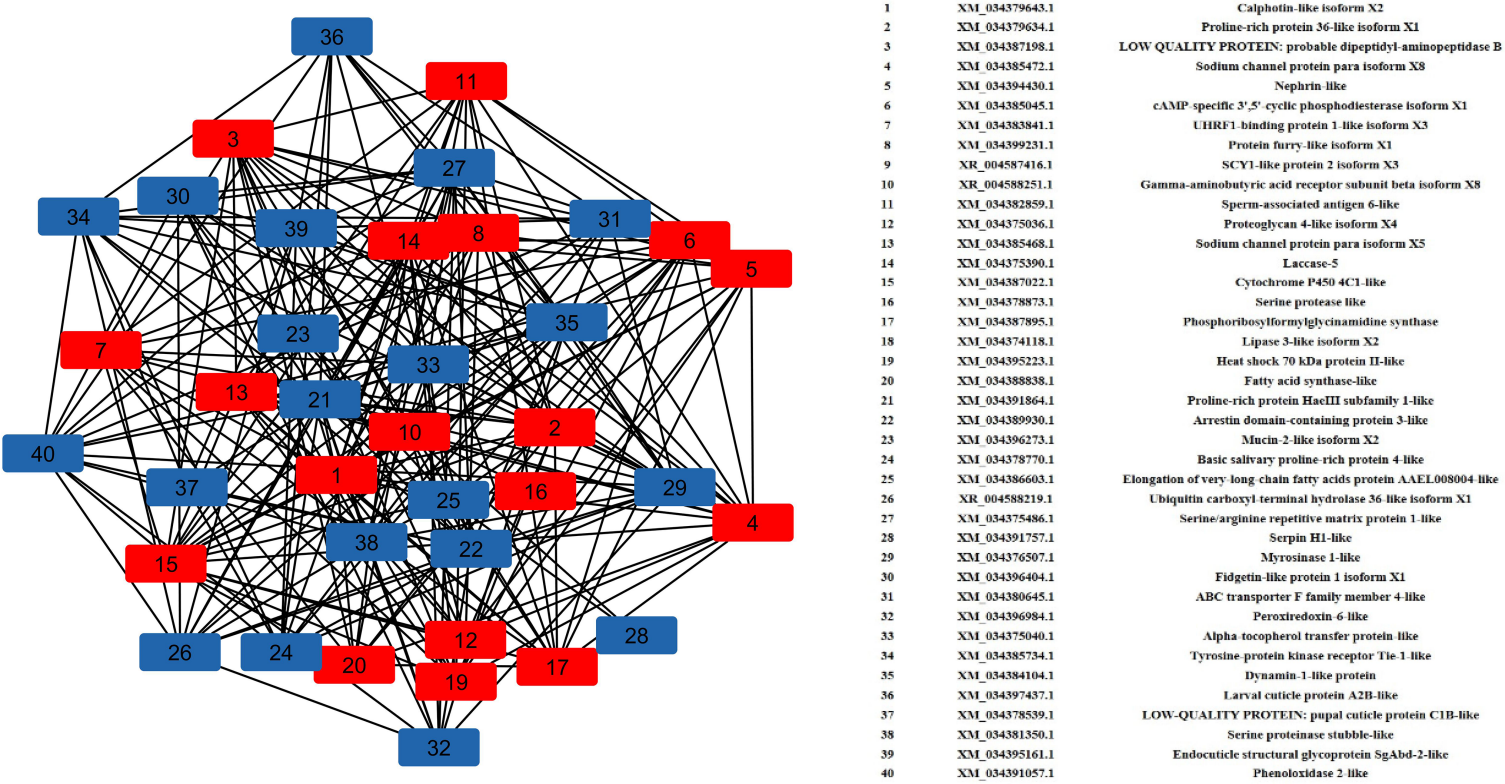
Gene regulatory network (GRN) of DEGs. Red squares represent upregulated DEGs, and blue squares represent downregulated DEGs.

A total of 40 nodes comprised the top up- and downregulated DEGs were considered in network analysis. The degree and betweenness centrality in the interaction network of the top DEGs were calculated. Interaction networks corresponding with the highly regulated DEGs demonstrated a total of 438 edges. The degree of certain nodes was markedly higher compared with the average degree in the networks (Figure 4). The upregulated DEGs with significantly higher degrees were *calphotin-like isoform X2, proline-rich protein 36-like isoform X1, dipeptidyl-aminopeptidase B, sodium channel protein para isoform X8, nephrin-like, cAMP-specific 3′,5′-cyclic phosphodiesterase isoform X1, UHRF1-binding protein 1-like isoform X3, spag6*-*like*, *laccase-5*, *CYP 4C1*-*like*, *phosphoribosylformylglycinamidine synthase* (*PFAS*), and *hsp70 II-like*. Among the downregulated nodes, a group of DEGs associated with cuticle development such as *larval cuticle protein A2B-like*, *endocuticle structural glycoprotein SgAbd-2-like*, and *ABC transporter F family member 4-like* were with higher degrees of interactions. Besides, *proline-rich protein HaeIII subfamily 1-like*, *ARRDC3*, *serpin H1-like*, *TIE-1-like*, *myrosinase 1-like*, *alpha-tocopherol transfer protein (TTPA)-like*, and *dynamin-1-like* protein showed significant interactions with higher degrees.

### Validation of DEGs in RT-qPCR

To validate the differential gene expression data, thirteen highly expressed genes of *T. palmi* were selected and mRNA expression levels were quantified in RT-qPCR in response to GBNV infection. A part of the samples used for RNA-Seq was preserved and used in RT-qPCR assay. Primer pairs designed for this study were initially optimized in a gradient PCR (Supplementary Table 1). Primer pairs of target genes that produced a single sharp amplicon at the same PCR conditions for endogenous control (*β-tubulin*) primers were selected for RT-qPCR assay. The primer pair specific to *β-tubulin* produced sharp bands at all annealing temperatures between 53 and 59°C. The annealing temperature of primer pairs for each target gene was standardized within the same temperature range. One best primer pair for each of the target genes was optimized for RT-qPCR assay. The annealing temperature, amplicon size, and melting temperature of each amplicon in RT-qPCR are included in Supplementary Table 1. The primer pairs for target and endogenous control genes produced single specific peaks without any secondary amplification in the RT-qPCR melting curve analysis that indicated the specificity of the reactions (Supplementary Figure 1).

**Figure 5:**
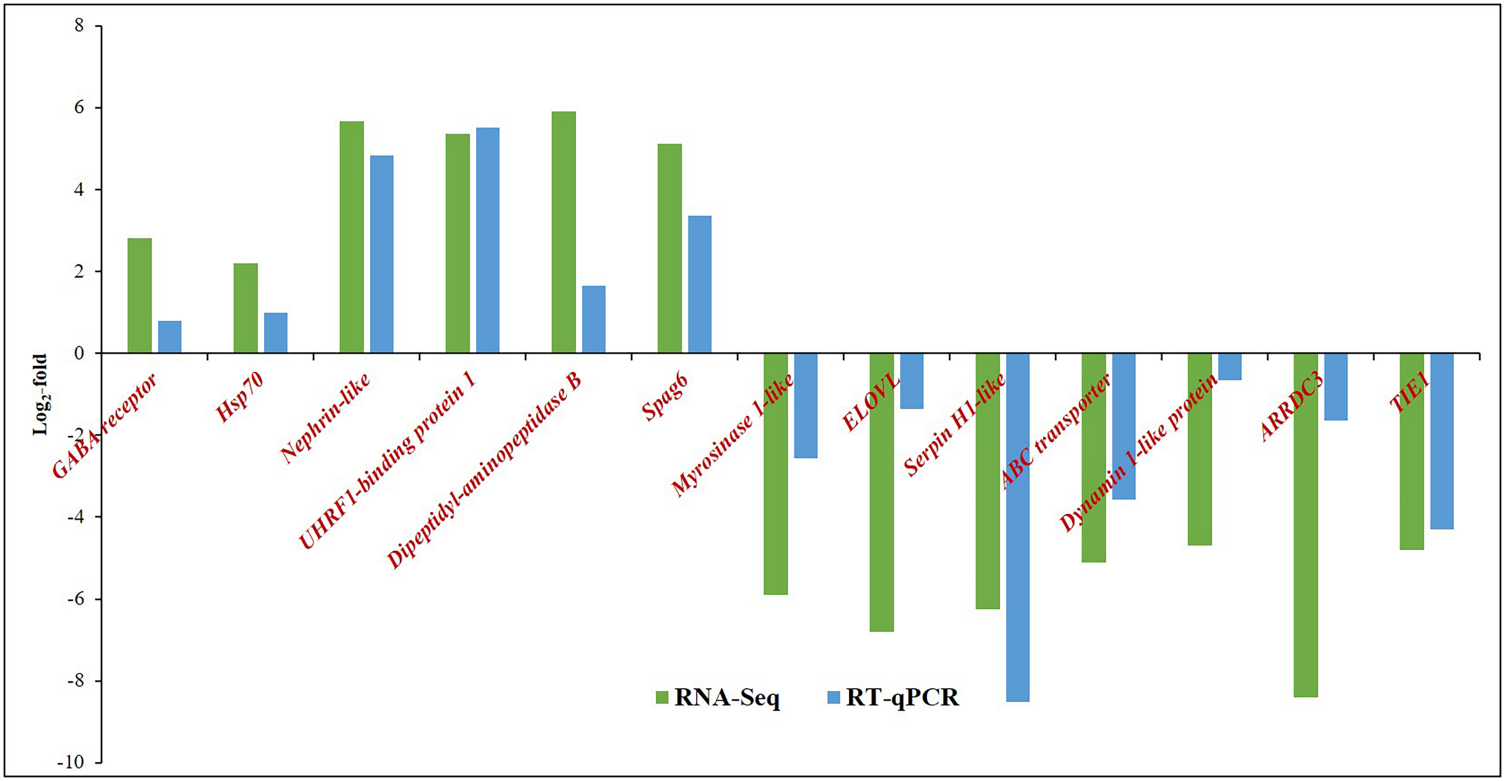
Expression of *Thrips palmi* putative genes in response to GBNV infection in RNA-Seq and reverse transcriptase-quantitative real-time PCR (RT-qPCR). The values of log_2_-fold changes calculated in RNA-Seq analysis were in accordance with the RT-qPCR fold change values. Pearson’s correlation coefficient value was 0.81 as calculated using the CORREL function in MS Excel.

The relative expression of target genes was estimated by a 2^−ΔΔCT^ method. The variation among the biological replicates was normalized with respect to the C_T_ value of endogenous control, *β-tubulin*. Among the selected highly expressed genes, the expression of *UHRF1-binding protein 1* of *T. palmi* was upregulated by log_2_ 5.5-fold in RT-qPCR in response to GBNV infection (Figure 5). Likewise, mRNA expression of *nephrin* was upregulated by log_2_ 4.8-fold in viruliferous *T. palmi* adults in comparison to nonviruliferous adults. mRNA levels of *spag6-like*, *hsp70*, and *GABA receptor* were upregulated by log_2_ 3.37, 0.98, and 0.80-fold, respectively, post-GBNV exposure. In viruliferous *T. palmi*, the expression of *serpin* was downregulated by log_2_ 8.5-fold. Similarly, log_2_ 4.3-fold downregulation of *tyrosin kinase* was recorded in RT-qPCR in response to GBNV infection. Expression of *ABC transporter F family member 4-like* gene was also downregulated by log_2_ 3.57-fold, while it was log_2_ 1.65-fold downregulations for *ARRDC3* in viruliferous *T. palmi*. Some other genes such as *elongation of fatty acid chain, dynamin-1-like, myrosinase 1-like* showed log_2_ 1.36, 0.66, and 2.57-fold, respectively, lower expression in viruliferous *T. palmi* as compared to nonviruliferous.

The fold change values estimated from RNA-Seq were plotted against fold change values obtained in RT-qPCR assays of the selected genes (Figure 5). The Pearson’s correlation coefficient of 0.81 indicated the acceptability of DEGs through RNA-Seq (Supplementary Table 3).

## Discussion

Multiplication of GBNV in *T. palmi* suggests a potential alteration in the physiological process of *T. palmi*. Pathogenic effects of GBNV are evident by reduced survival and adult longevity of *T. palmi* (Ghosh et al., 2019). The fecundity of *T. palmi* decreases sharply after GBNV exposure. GBNV infection in *T. palmi* also induces more females in progenies (Ghosh et al., 2019). We observed the same trend in the biological traits of *T. palmi* post-GBNV exposure in the current study. In thrips–tospovirus relationships, adults of thrips are vector competent only if the virus is acquired during the larval stages (Wetering et al., 1996; Mou et al., 2021). Only during the early larval stage, the virus is allowed to infect the midgut (Ullman et al., 1992; Ohnishi et al., 2001). The virus infects the epithelial cells of the anterior midgut of *T. palmi* and then progresses to the primary salivary glands through a pair of connecting ligaments (Ghosh et al., 2021). The tospovirus infection in adults of *T. palmi* is retained in visceral and longitudinal muscles of the midgut. Tospovirus may not be able to infect the epithelial cells of adult thrips (Ghosh et al., 2021). A 50-kDa protein receptor present on the epithelial cells of thrips is abundant during the larval stage only (Bandla et al., 1998). The tospovirus propagates in the midgut and salivary glands of *T. palmi* (Ghosh et al., 2021; Mou et al., 2021). In thrips, a transovarial infection barrier has been discovered, which prevents the tospovirus from transmitting vertically (Wijkamp et al., 1996; Mou et al., 2021). The next generation of thrips again needs to acquire the virus during their early larval stage.

Manipulation of insect vectors by plant viruses is common in thrips–tospovirus systems (Deangelis et al., 1993; Wijkamp et al., 1996; Belliure et al., 2008; Ingwell et al., 2012; Shrestha et al., 2012; Ghosh et al., 2019). The central hypothesis of the present study tells that the successful GBNV transmission by viruliferous *T. palmi* perturbs the expression of candidate genes associated with innate immunity, cuticle development, cellular receptor binding, and signaling of its vector. The differential transcriptomic response of *T. palmi* induced by GBNV infection was distinguished by RNA-Seq. KEGG analyses further explained several molecular pathways involved in *T. palmi*-GBNV interactions. Cluster analysis of expressed genes from viruliferous and nonviruliferous *T. palmi* revealed that 1,383 and 980 genes of *T. palmi* were up- and downregulated, respectively in response to GBNV infection. TSWV infection regulated expression of 127 transcripts in *F. occidentalis* adults. Expression of genes like *arylphorin*, *chitinase*, *hemocyanin*, and *hexamerin* was modulated in *F. occidentalis* adults in response to TSWV infection (Schneweis et al., 2017). A total of 562 contigs were differentially regulated by TSWV in adults of *F. fusca*. Reproduction, embryo development, and growth-related pathways were identified with upregulated contigs in virus-exposed adults (Shrestha et al., 2017). In the present study, candidate genes, such as *CYP*, *GABA receptor*, *hsp70*, *laccase 5*, *nephrin*, *spag6*, *PFAS*, *proteoglycan*, and *UHRF1-binding protein 1* associated with innate immune response, receptor binding, signaling, endocytosis, viral replication, and apoptosis (Qin et al., 2009; González-Santoyo and Córdoba-Aguilar, 2012; Götz et al., 2012; Cooley et al., 2016; Zhang et al., 2016; Zhu et al., 2017; Lu et al., 2019) were upregulated in *T. palmi* adults in response to GBNV infection.

A group of genes associated with host innate immune and stress response, such as *hsp70*, *UHRF1-binding protein 1*, *spag6*, *laccase-5*, and *PFAS* was upregulated in viruliferous *T. palmi* (Zhao and Jones, 2012; Cooley et al., 2016; Zhang et al., 2016; Chen et al., 2020). *Hsp* belongs to a class of functionally related proteins involved in the folding and unfolding of other proteins. This group of proteins is elevated in insect cells in response to various biotic and abiotic stresses (Zhao and Jones, 2012). Besides, *hsp70* is an active regulator of tomato yellow leaf curl virus (TYLCV) infection in its vector, *Bemisia tabaci* (Götz et al., 2012). In infected host cells, *hsp70* interacts with TYLCV coat protein (CP) and is involved in the translocation of TYLCV CP from the cytoplasm to nucleus (Götz et al., 2012; Gorovits and Czosnek, 2013, 2017). Upregulations of *T. palmi hsp70* were also recorded in response to CaCV infection (Gamage et al., 2018). However, interaction of *hsp70* with GBNV surface proteins needs further experimental evidence. *UHRF1-binding protein 1* is best known for its function in maintaining DNA methylation and acting as an epigenetic regulator. When the host is attacked by pathogens, gene expression levels are regulated by a DNA methylation pattern that leads to activation or suppression of several signaling pathways and triggers a series of immune response events against viral invasion (Zhang et al., 2016). Thus, upregulation of *UHRF1-binding protein 1* in viruliferous *T. palmi* adults suggests its role in innate immunity. Upregulation of *spag6* in the present study might also be due to the immune response of *T. palmi* to GBNV infection. The role of mouse *spag6* in immune response was described by Cooley et al. (2016). In insects, laccase is abundant in the cuticle and has been involved in cuticle sclerotization (Hattori et al., 2010). *Laccase 5* was significantly upregulated in viruliferous *T. palmi*. *Laccases* include phenol oxidases (Janusz et al., 2020) that are activated in response to viral infection and oxidative stress (González-Santoyo and Córdoba-Aguilar, 2012; Chen et al., 2020). A similar response of *T. palmi laccase* post-CaCV infection (Gamage et al., 2018) suggests its association in maintaining oxidative stress due to tospovirus infection in *T. palmi*. In consistent with the previous report of CaCV-exposed *T. palmi* (Gamage et al., 2018), *PFAS* was also upregulated in *T. palmi* in response to GBNV infection. *PFAS* was also found to be upregulated in *Culex quinquefasciatus* after infection with West Nile virus (Girard et al., 2010). *PFAS* not only plays a role in the immune escape process of the virus but also has a crucial impact on other pathological processes (Lu et al., 2019). Pathological effects such as reduced survival and adult longevity of *T. palmi* were also recorded post-GBNV exposure in the present study. The role of *PFAS* in triggering such pathological effects in *T. palmi* may be worth for further investigation.

The GBNV is supposed to be internalized in *T. palmi* cells by clathrin-mediated endocytosis (CME; Jagdale and Ghosh, 2019). DEGs, such as *nephrin*, *proteoglycan 4-like*, and *heparin sulfate proteoglycan* (*HSPG*) associated with endocytosis and entry receptors (Qin et al., 2009; Christianson and Belting, 2014; Cagno et al., 2019) were upregulated upon GBNV infection. *Nephrin* is internalized during the signal transduction pathway of raft-mediated endocytosis and classical CME (Qin et al., 2009). The upregulation of *nephrin* in the present study indicated its involvement in CME of the GBNV proteins in *T. palmi* cells. *Proteoglycan 4-like*, upregulated in *T. palmi* post-GBNV infection, might be associated with receptor binding and entry of tospovirus in thrips. *HSPG* is a cell-surface endocytosis receptor and is involved in cellular interactions with several human viruses. The interaction with *HSPG* is used by viruses to increase their concentration at the cell surface and augment their chances of binding more specific entry receptors (Cagno et al., 2019). *HSPG* is an integral factor for the attachment of Arbovirus to the salivary gland duct in mosquitoes (Ciano et al., 2014).

In insects, *GABA receptors* are found throughout the central nervous system and are reported to be an important target site for several insecticides (Anthony et al., 1993). In mosquitoes, *GABA receptor* was associated with viral replication (Zhu et al., 2017). *CYP* imparts resistance against insecticides in *F. occidentalis* and *T. tabaci* (Zhang et al., 2013; Rosen et al., 2021). In the present study, upregulations of *GABA receptor* and *CYP* in viruliferous *T. palmi* were in accordance with the response of CaCV-exposed *T. palmi* (Gamage et al., 2018). *CYP*-mediated metabolism of xenobiotics pathway was also enriched in viruliferous *T. palmi*. An in-depth study of the mechanism involved would be necessary to shed further light on the role of *GABA receptor* and *CYP* in tospovirus replication in *T. palmi*.

A considerable number of *T. palmi* genes were under-regulated in response to GBNV infection. Downregulations of genes like *ABC transporter*, *ARRDC3*, *cuticular proteins*, *dynamin-1-like protein*, *myrosinase-1-like*, and *serpin* facilitate the circulation and multiplication of GBNV in *T. palmi*. Several cuticular protein genes were downregulated in transcriptomic studies of TSWV-infected *F. occidentalis* (Schneweis et al., 2017) and CaCV-infected *T. palmi* (Gamage et al., 2018). In the present study, downregulations of *T. palmi larval cuticle protein A2B-like*, *pupal cuticle protein C1B-like*, *endocuticle structural glycoprotein SgAbd-2-like* were recorded in response to GBNV infection. *Endocuticle structural glycoprotein* localizes in the midguts and salivary glands of *F. occidentalis* (Schneweis et al., 2017). *ABC transporter* has also been reported to be involved in molting and cuticle formation in insects (Broehan et al., 2013) and was downregulated in the present study. Virus infection suppresses the activities of cuticular proteins embedded in the peritrophic matrix, a structural barrier to pathogen attack (Kato et al., 2008; Levy et al., 2011). In consistent with the downregulation of genes associated with cuticle development, a delayed molting of the larval instars post-GBNV exposure was recorded in the present study. Delayed molting of viruliferous *T. palmi* was earlier reported by Ghosh et al. (2019). Under-expression of *cuticular*, *endo-cuticular proteins*, and *ABC transporter* of *T. palmi* gives an advantage of the delayed molting and membrane dysfunction for virus infection in the thrips gut. However, insect cuticle-related contigs were upregulated in TSWV-infected *F. fusca* (Shrestha et al., 2017). The innate differences among the thrips species, experimental setup, and virus isolates could be the reason behind the variation in response.

A few genes associated with innate immune responses like *serpins* and *dynamin* were also downregulated in *T. palmi* in response to GBNV infection. Insect innate immunity is regulated by *serpins* by inhibiting serine proteinase cascades that instigate innate immune responses. *Serpins* could also possess direct anti-pathogen activity upon infection (Levashina et al., 1999). *Dynamin-1-like protein* is close relative to *dynamin* and has a role in fission of mitochondrial and peroxisomal membranes. In mammals, mitochondrial dynamin affects disease resistance. Pathogens have evolved several strategies to interfere with mitochondrial dynamins to suppress the immune responses (Spier et al., 2019). Several pathogens directly target the *dynamin-related protein (DRP1)* to block the innate immune responses (Suzuki et al., 2014; Yu et al., 2015). Downregulation of *dynamin-1-like protein* and *serpin H1-like* genes in viruliferous *T. palmi* help in evading innate immune responses in *T. palmi* to favor the virus infection and multiplication in vector cells. Gene like *ELOVL* was downregulated in viruliferous *T. palmi*. Accumulation of ELOVL fatty acids in the cells leads to necroptosis (Parisi et al., 2017). Inhibition of *ELOVL* prevents the loss of plasma membrane integrity and cell death. Downregulation of *ELOVL* gene in *T. palmi* helps maintain the integrity of cells and supports viral growth.

A group of genes associated with cell surface receptor and cellular transport, such as *TIE1*, *ARRDC3*, *TTPA-like*, and *potassium channel subfamily K member 18 (KCNK18)-like* was regulated in *T. palmi* in response to GBNV infection. *TIE1* is the major class of enzyme-coupled cell surface receptors activated in response to external signals such as growth factors, cytokines, and hormones. Host signaling through *tyrosine receptor kinases* has also been found to play a key role in virus replication (Kumar et al., 2011). Recently, it has been demonstrated that *tyrosine kinases* are required by viruses for intracellular entry (Stewart et al., 2021). *ARRDC3* is a member of the family of α-arrestins in the superfamily of arrestin adaptor proteins (Patwari and Lee, 2012). The α-arrestin, *ARRDC3* mediates ALIX ubiquitination and G protein-coupled receptor lysosomal sorting (Dores et al., 2015). Ubiquitination of ALIX has also been observed during the budding of viruses from the plasma membrane in mammals (Sette et al., 2010). *TTPA*-*like* and *KCNK18-like* that encode proteins associated with inter- and intracellular transport were downregulated in response to GBNV infection. Similar results were recorded by Gamage et al. (2018). *TTPA isoform 1* and *2*, and *KCNK9* were also downregulated in response to TSWV infection in *F. fusca* (Shrestha et al., 2017). The requirement of potassium channels for infecting the host cell by bunyaviruses was reported by Hover et al. (2016). The antiviral activity of the potassium ion channel in honeybees was reported by O’Neal et al. (2017). However, the molecular mechanisms underlying the downregulation of these genes in response to GBNV infection are not known and need in-depth study.

The comparison of the DEGs reported in CaCV-exposed *T. palmi* (Gamage et al., 2018) with our work showed 103 functions in common (Supplementary Figure 2). However, we could find 2,260 genes uniquely expressed in the GBNV-exposed *T. palmi*, while 347 genes were unique to the CaCV-exposed *T palmi*. Among the DEGs, the responses of *cuticular proteins*, *CYP*, *endocuticle structural glycoprotein*, *GABA receptor*, *hsp70*, *laccase 5*, *KCNK-like*, and *TTPA* were conserved in both GBNV and CaCV infection but distinct to TSWV infection in *F. occidentalis* (Schneweis et al., 2017). These candidate genes would be potential targets for novel generic pest control.

Among the major pathways of *T. palmi* affected by GBNV, metabolic pathways, biosynthesis of secondary metabolites, endocytosis, and signaling pathways were important. Ascorbate and aldarate metabolisms, steroid hormone biosynthesis, cytochrome p 450, and other enzymes-mediated metabolism of xenobiotics, fructose, and mannose metabolism were highly enriched. GRN analysis showed that DEGs associated with innate immune response, cuticle development, receptor signaling, and endocytotic pathways are involved in possible manipulation in host invasion by GBNV.

In conclusion, we have assembled a whole-body transcriptome of adult *T. palmi* and reported the DEGs in response to GBNV infection. The majority of DEGs are involved in innate immunity, receptor binding and signaling, cuticle development, and endocytosis that facilitate the invasion, circulation, and multiplication of GBNV in *T. palmi*. The candidate genes of *T. palmi* that are highly regulated in response to GBNV infection would be potential targets for the effective management of tospoviruses. In addition, data generated in this study will enrich genomic information of thrips and will enable functional studies.

## Data Availability Statement

The datasets presented in this study can be found in online repositories. The names of the repository/repositories and accession number(s) can be found at: https://www.ncbi.nlm.nih.gov/, PRJNA758768.

## Author Contributions

AG, VB, VK, and SC conceived and designed the research. DM prepared biological samples. DM, SJn, and Priti carried out the wet laboratory experiments and wrote the draft manuscript. Priti, MI, and SJi analyzed RNA-Seq data. AG and VB reviewed the results and edited the final manuscript. All authors read and approved the manuscript.

## Funding

The scholarship of DM was supported by Indian Council of Agricultural Research.

## Conflict of Interest

The authors declare that the research was conducted in the absence of any commercial or financial relationships that could be construed as a potential conflict of interest.

## Publisher’s Note

All claims expressed in this article are solely those of the authors and do not necessarily represent those of their affiliated organizations, or those of the publisher, the editors and the reviewers. Any product that may be evaluated in this article, or claim that may be made by its manufacturer, is not guaranteed or endorsed by the publisher.

## Footnotes

^1^ https://www.bioinformatics.babraham.ac.uk/projects/trim_galore/

^2^ http://www.bioconductor.org/packages/release/bioc/html/DESeq.html

^3^ http://geneontology.org/

^4^ https://www.ebi.ac.uk

^5^ http://www.genome.jp/kegg
