# Supplementary material for: Groundnut Bud Necrosis Virus Modulates the Expression of Innate Immune, Endocytosis, and Cuticle Development-Associated Genes to Circulate and Propagate in Its Vector, Thrips palmi
Source: Front Microbiol. 2022 Mar 17;13:773238. doi: 10.3389/fmicb.2022.773238 (PMC8969747; doi:10.3389/fmicb.2022.773238)
Supplement: Supplementary file 1 [file Data_Sheet_1.pdf]

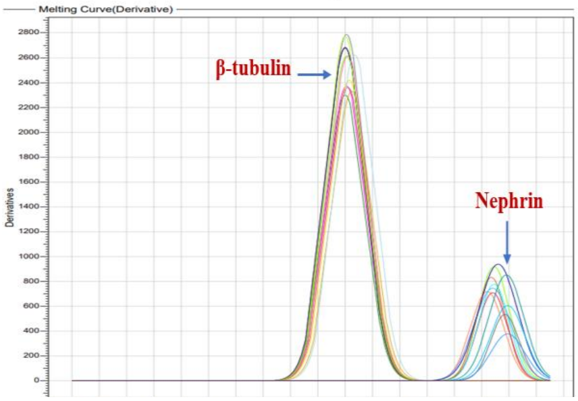

1. Nephrin

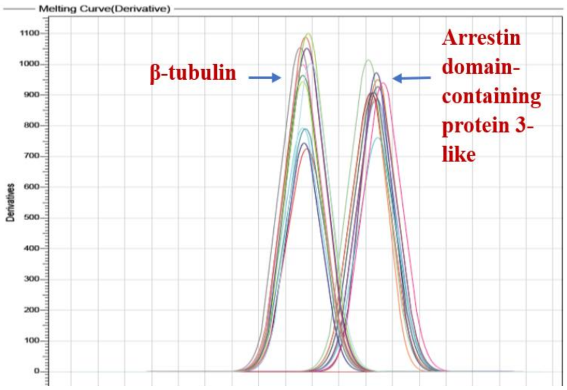

2. Arrestin domain-containing protein 3-like

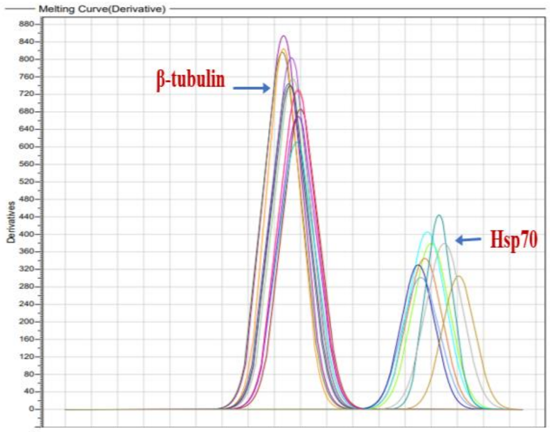

3. Heat shock protein 70

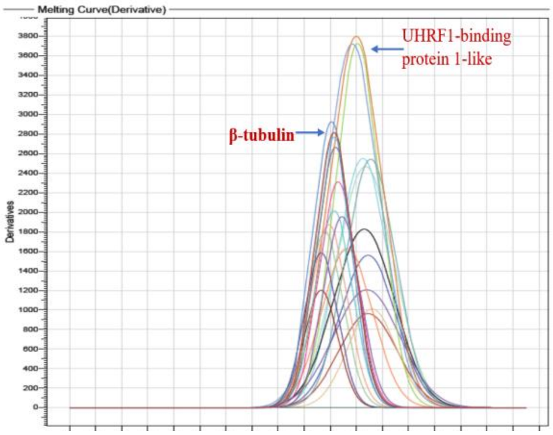

4. UHRF1-binding protein 1-like

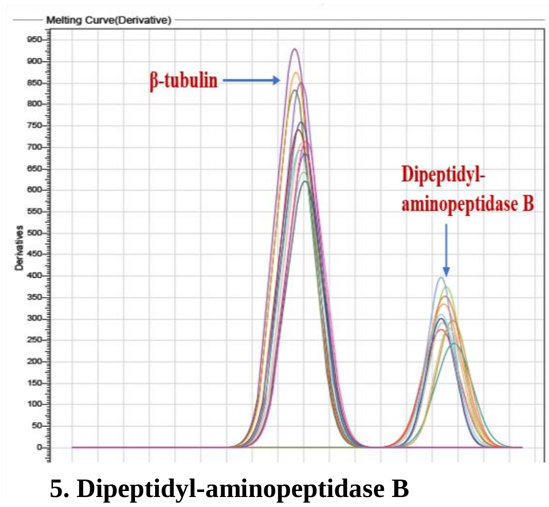

5. Dipeptidyl-aminopeptidase B

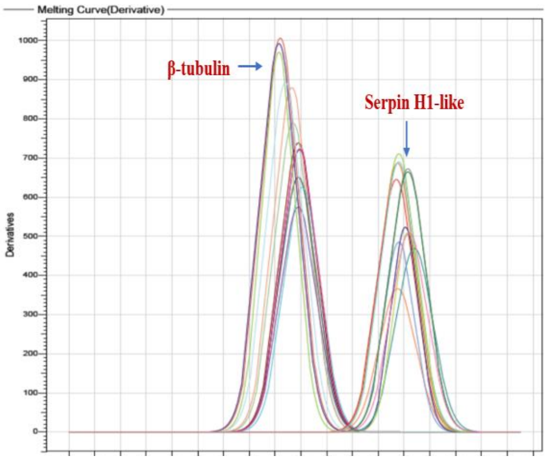

6. Serpin H1-like

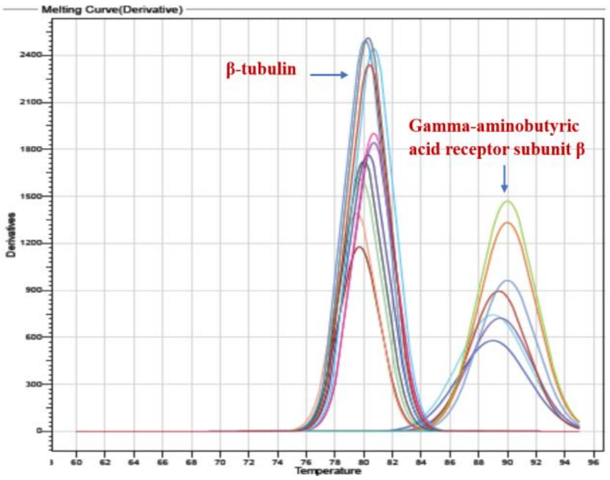

7. Gamma-aminobutyric acid receptor subunit beta

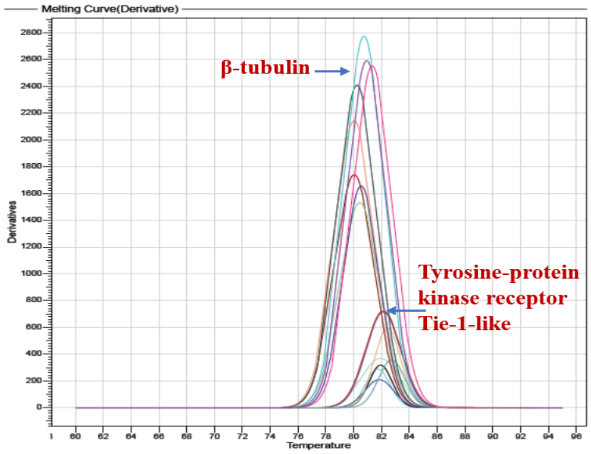

8. Tyrosin-protein kinase receptor Tie 1-like

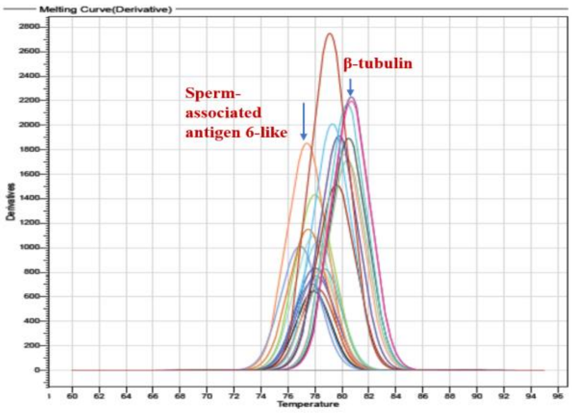

9. Sperm associated antigen 6-like

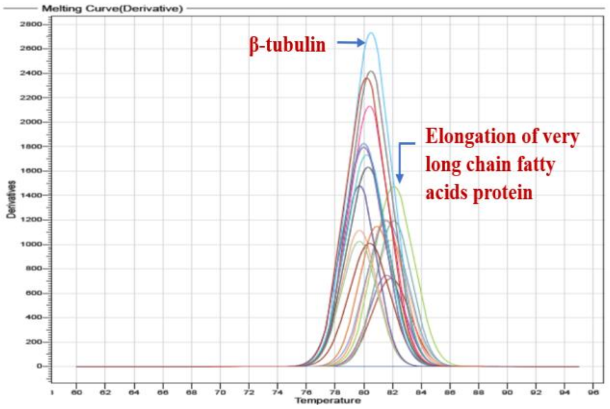

10. Elongation of very long chain fatty acids protein

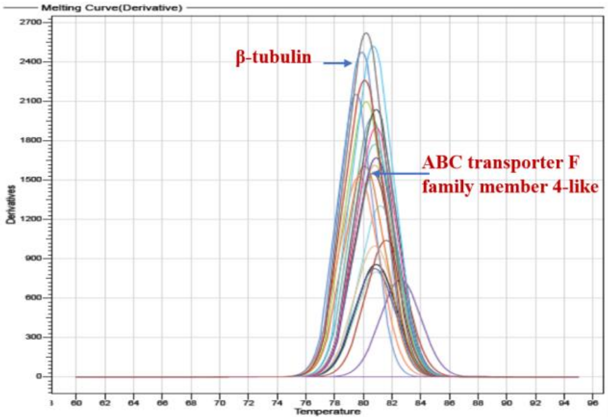

11. ABC transporter F family member 4-like

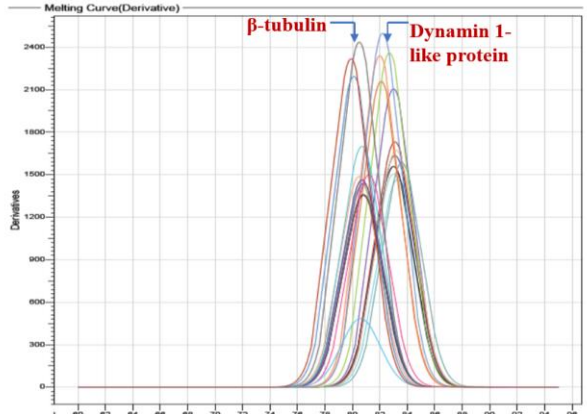

12. Dynamin 1-like protein

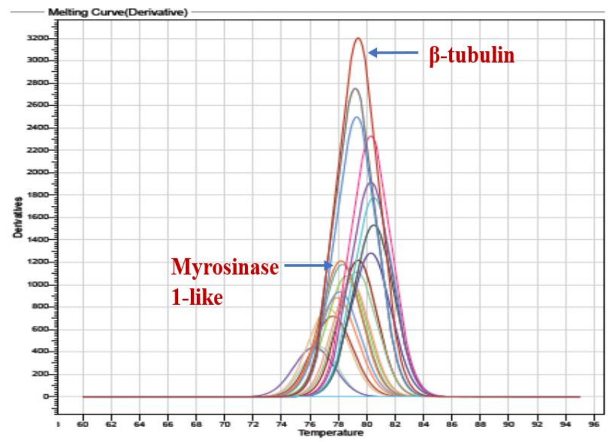

Supplementary figure 1. Melting curves of target and endogenous control genes of *Thrips palmi* in qRT-PCR

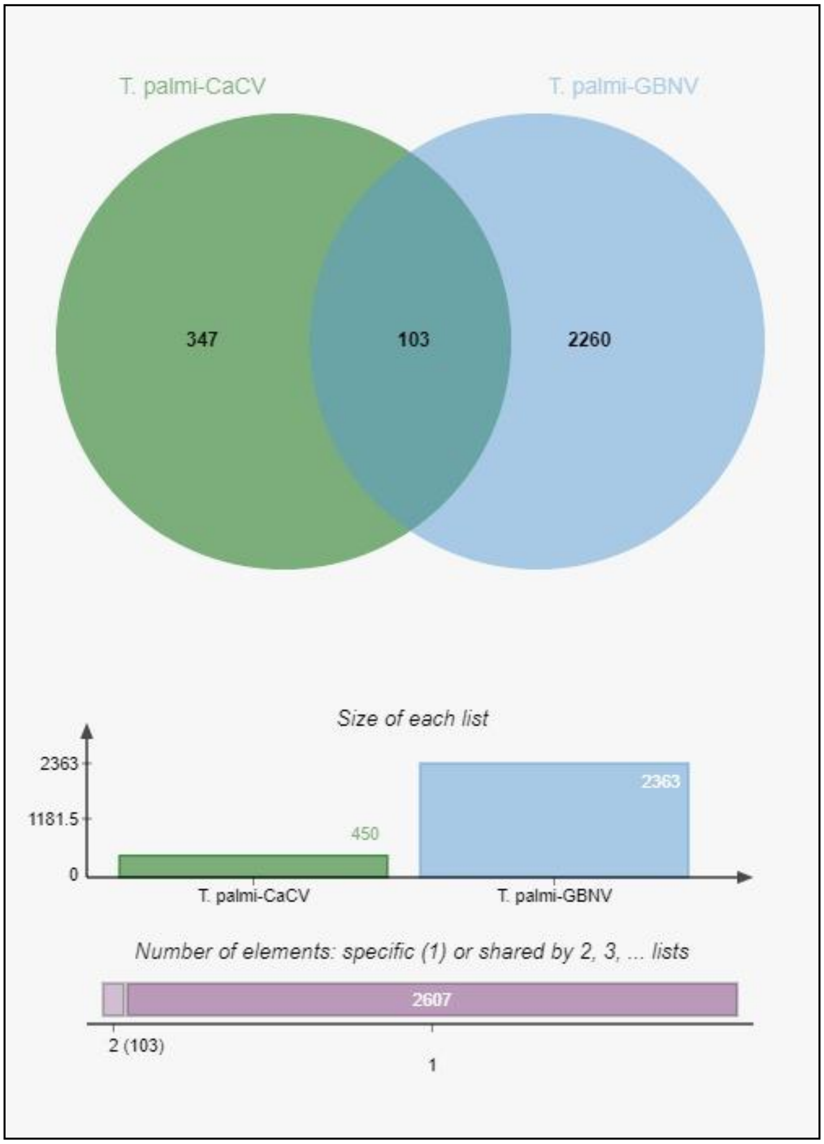

Supplementary figure 2. Venn diagram of DEGs of *T. palmi* in response to GBNV and CaCV infection. A total of 103 DEGs are conserved in response to GBNV and CaCV infection.

Supplementary Table 1. List of primers used in the study

S. No.	Gene name	Primer Name	Primer Sequence	Annealing temperature (°C) in PCR	Amplicon size (bp)	Melting temperature (°C) in RT-qPCR	References
**Primer used for *Thrips palmi* identification in PCR**							
1	Mt Cox I region, universal primer	LCO1490	GGTCAACAAATCATAAAGATATTGG	50	657	-	Folmer et al., 1994
		HC02198	TAAACTTCAGGGTGACCAAAAAATCA				
**Primer used for Groundnut bud necrosis virus diagnosis in PCR**							
2	GBNV N gene	AG109-F	CCATCTACTTCAGTAGAAAACACTAG	59	1767	-	This study
		AG110-R	AGAGCAATCAGTGCAACAATTAAATA				
**Primers used for *Thrips palmi* gene expression study in qRT-PCR**							
3	ß- tubulin (endogenous control)	AG171-F	CCAGCCACATTCCTGGATAC	53-59	117	79.5	Gamage et al. 2018
		AG172-R	ATGCGTTGGCAGTCACATAC				
4	Gamma-aminobutyric acid receptor	AG347-F	CGGTTCATAGTTCACGACCC	55	221	89.4	This study
		AG348-R	ATGAGGTTGAAGCACACGAA				
5	Heat shock 70 kDa protein II-like	AG353-F	GTATGGTACCAGGGGAAGGT	59	156	88.3	This study
		AG354-R	CTTTGCGTCGAACACGGT				
6	Dipeptidyl-aminopeptidase B	AG361-F	CGGTACCCGAAGATCAACAC	53	163	89.1	This study
		AG362-R	AGTAGTAGTCGGACTCCCTG				
7	Nephrin-like	AG363-F	CCGAGAAGCAGACTGAAGAG	59	189	91.1	This study
		AG364-R	AATCCTTGTTGCAGGGGATG				
8	Sperm-associated antigen 6-like	AG375-F	GACAAGAAGGAGACGTGGCT	55	171	77.9	This study
		AG376-R	AGACAGCACCGAGGGTTTG				
9	UHRF1-binding protein 1	AG379-F	CGGAGGTGCTCTTCAAATCA	55	190	82.7	This study
		AG380-R	AACTGCACGGTTGCTTTCTA				
10	ABC transporter	AG383-F	GAGGAAGCGTGAACCATCTG	55	159	80.6	This study

		AG384-R	TCCTCCTCATGCTGATCTGT				
11	Arrestin domain-containing protein	AG389-F	GAGTCTTCCTGAACGTGAGC	54	113	82.7	This study
		AG390-R	GCCGTGACTGTTAGACTTGG				
12	Dynamin-1-like protein	AG399-F	CGTAGTCGCTAAAACCGTCA	55	173	82.8	This study
		AG400-R	TAGTTTCGGCTCTCAGTTGC				
13	Elongation of very long chain fatty acids protein	AG403-F	CGAATGGATACCACACCCAC	55	171	86.2	This study
		AG404-R	GTGGTTCAATCCTGTACGCA				
14	Myrosinase 1-like	AG411-F	TCTACGAGCGCGAGTTTAAG	55	188	80.2	This study
		AG412-R	GTTCCTCTTCATGAGCTCGG				
15	Serpin H1-like	AG431-F	GAGTTCCCCGGTATCTCCAA	59	183	85.88	This study
		AG432-R	GCGCAGGTAGAAGACAAAGG				
16	Tyrosine-protein kinase receptor Tie-1-like	AG433-F	CAGCAAGGTATAACGCACCT	55	114	81.6	This study
		AG434-R	RGACAGAGCCTTCTCCTTTCG				

Supplementary Table 2. Summary statistics of generated RNA-Seq data, before and after pre-processing along with the mapping percentage with the reference genome of *Thrips palmi*

Sample	Raw reads	Filtered reads	% of reads mapped with reference genome
TpTrH1	24463812	24452353 (99.95%)	97.65
TpTrH2	25960270	25947964 (99.95%)	97.80
TpTrH3	25490048	25478767 (99.95%)	97.72
TpTrI1	24893045	24885567 (99.96%)	96.22
TpTrI2	26320266	26316161 (99.98%)	97.34
TpTrI3	29000000	28993257 (99.97%)	97.11

Supplementary Table 3. Relative expression of *T. palmi* candidate genes in RNA-Seq and RT-qPCR

Sl. No.	Candidate gene	Relative expression in Log_2 fold change		Remarks
		RNA-Seq (P-adj)	RT-qPCR	
1.	UHRF1-binding protein 1-like isoform X3	5.35 (8.31E-49)	5.5	Upregulated
2.	Nephrin-like	5.68 (1.39E-10)	4.8	
3.	Sperm-associated antigen 6-like	5.12 (1.07E-06)	3.37	
4.	Heat shock 70 kDa protein II-like	2.22 (6.00E-07)	0.98	
5.	Gamma-aminobutyric acid receptor subunit beta isoform X8	5.14 (4.51E-07)	0.80	
6.	LOW-QUALITY PROTEIN: probable dipeptidyl-aminopeptidase B	5.99 (2.31E-42)	1.64	
7.	Serpin H1-like	-6.25 (1.98E-99)	8.5	Downregulated
8.	Tyrosine-protein kinase receptor Tie-1-like	-4.88 (1.37E-111)	4.3	
9.	ABC transporter F family member 4-like	-5.11 (2.06E-20)	3.57	
10.	Arrestin domain-containing protein 3-like	-8.96 (1.73E-196)	1.65	
11.	Elongation of very-long-chain fatty acids protein AAEL008004-like	-6.80 (2.61E-32)	1.36	
12.	Dynamin-1-like protein	-4.73 (4.80E-185)	0.66	
13.	Myrosinase 1-like	-5.92 (9.55E-222)	2.57	

The relative expression of candidate genes was estimated by comparing three sets of viruliferous and three sets of non-viruliferous *T. palmi* populations. The statistical significance of log_2 fold change in RNA-Seq was assessed using adjusted P values taking into account the false discovery rate (FDR). The adjusted P values are mentioned in parenthesis. The fold-change in RT-qPCR was estimated by $2^{-\Delta\Delta CT}$ method (Livak and Schmittgen 2001). The ΔCT was calculated as CT of targeted gene - CT of endogenous control. ΔΔCT value was calculated by average ΔCT of non-viruliferous replicates - ΔCT of each replicate. The average $2^{-(\Delta\Delta CT)}$ was calculated for both the viruliferous and non-viruliferous replicates and transformed to $log_2\ 2^{-(\Delta\Delta CT)}$. The log_2-fold change of the gene expression in the viruliferous *T. palmi* population was estimated by normalizing the values. The correlation of RNA-Seq and RT-qPCR values was calculated using CORREL function in MS Excel. Pearson's correlation coefficient value was 0.81.
